# Exploring the mechanism of anti-fatigue of resveratrol based on network pharmacology and molecular docking, and in vitro studies

**DOI:** 10.1038/s41598-023-30141-w

**Published:** 2023-02-18

**Authors:** Peipei Ma, Jinlei Li, Qing Huang, Shijie Wei, Hurong Ge, Zhizhong Wang

**Affiliations:** 1 School of Basic Medicine, Ningxia Medical University, Yinchuan, 750004 Ningxia China; 2 School of Pharmacy, Ningxia Medical University, Yinchuan, 750004 Ningxia China; 3 Department of Pharmacy, General Hospital of Ningxia Medical University, Yinchuan, 750004 Ningxia China; 4 School of Sports, Ningxia Medical University, Yinchuan, 750004 Ningxia China

**Keywords:** Computational biology and bioinformatics, Medical research

## Abstract

To investigate the potential mechanism of resveratrol in anti-fatigue by network pharmacology and molecular docking, and to investigate the anti-fatigue efficacy of resveratrol through in vitro animal experiments. Resveratrol action targets and fatigue-related targets were obtained using various databases. The anti-fatigue targets of resveratrol were obtained using the Venn diagram, uploaded to the String database, imported into Cytoscape 3.7.1, and constructed into a Protein-protein interaction network. The target genes were then subjected to Gene ontology and Kyoto encyclopedia of gene and genome enrichment analysis. Molecular docking verification was performed on the binding ability of the core target to resveratrol. Using swimming-trained mice as exercise models, exhaustive swimming time and fatigue-related biochemical parameters were used as indicators to investigate the effects of resveratrol on exercise endurance and energy metabolism. 104 anti-fatigue targets and 10 core target genes of resveratrol were obtained. KEGG analysis enrichment included AGE-RAGE signaling pathway in diabetic complications, Human cytomegalovirus infection, and Pathways in cancer. Molecular docking showed that the core target genes TP53, PIK3R1, AKT1, PIK3CA, and MAPK1 had good binding activity to resveratrol. Animal experiments showed that resveratrol could prolong the exhaustive swimming time of endurance-trained mice (P < 0.01), decrease aspartate aminotransferase, alanine aminotransferase, uric acid, blood lactate (P < 0.01), decrease blood urea nitrogen (P < 0.05), increase the liver glycogen, muscle glycogen (P < 0.01). Conclusion: Resveratrol has the characteristics of multiple targets and multiple pathways in anti-fatigue; resveratrol can enhance exercise endurance in mice.

## Introduction

Fatigue is a feeling of tiredness and powerlessness. Objectively, under the same conditions, it is impossible to complete the work and activities that can be done before^1^. Normal fatigue that occurs after intense physical exertion can be relieved by rest or lifestyle changes^2^. But pathological fatigue cannot be improved by rest^3^. Exercise-induced fatigue is the temporary reduction of human working ability caused by exercise. Although exercise-induced fatigue is a normal protective mechanism of the human body, if it is in a state of fatigue for a long time, it will cause irreversible damage to the body’s metabolism and normal exercise ability^4^. Due to the unsettlement of a series of problems that have been brought up by fatigue and exercise-induced fatigue, a highly concerned topic in the competitive sports area, the study on how to quickly alleviate fatigue and improve athletic performance has attracted a lot of attention from researchers^5^.Although ginseng, Ganoderma lucidum, and other traditional Chinese medicines have anti-fatigue effects, people are still searching for an effective, green, and harmless nutritional supplement that can resist exercise-induced fatigue.

Resveratrol, it has anti-tumor, antibacterial, anti-inflammatory, anti-aging, anti-viral, liver and kidney protection and immune regulation effects^6^ The in-depth research of resveratrol, found that it also has a significant effect on fatigue^7^. The anti-fatigue effect of resveratrol has also been previously reviewed in the literature^8^. A research team at the University of Alberta in Canada has demonstrated that resveratrol can improve sports training and performance^9^. However, the specific mechanism of action of resveratrol against fatigue remains unclear.

Network pharmacology is widely used in drug research because of its holistic, systematic, and efficient nature, using bioinformatics, molecular biology, and database systems to study the relationship between “drug-target-pathway-disease”^10,11^. Network pharmacology explores the relationship between drug components and targeted diseases from the perspective of systems biology, predicts the underlying mechanism of drug treatment of diseases, and provides a theoretical basis for the effectiveness of drug treatment of diseases^12^. Therefore, this study adopts the method of network pharmacology, collects data and information from multiple platforms, explores the potential mechanism of resveratrol in fatigue treatment, provides a theoretical basis for resveratrol’s anti-fatigue, and further through in vitro animal experiments to investigate the anti-fatigue effect of resveratrol.

## Methods

### Screening of the target of resveratrol

Resveratrol targets from CTD, DGIdb, DrugBank, Swiss Target Prediction, and TCMSP databases were obtained, merged, deduplicated targets, and eliminated non-human targets.

### Screening of the target of fatigue

Fatigue targets from GeneCard, DisGeNET, OMIM, and DrugBank databases. Merge to remove duplicate targets.

### Screening of anti-fatigue targets of resveratrol

The intersection of the resveratrol action target and fatigue target using the Venn diagram is the resveratrol anti-fatigue target.

### Construction of the resveratrol anti-fatigue target Protein-protein interaction (PPI) network and screening of the core target proteins

Upload the resveratrol anti-fatigue target proteins to the String database, and select protein interactions with a confidence score > 0.4. The obtained data were imported into Cytoscape 3.7.1, the PPI network of resveratrol anti-fatigue target protein was constructed, and the topological parameters moderate centrality, compact centrality, and betweenness centrality were analyzed. It is important to screen the top 10 proteins as core target proteins.

### Enrichment analysis of resveratrol anti-fatigue target genes

The DAVID database was used for enrichment analysis of resveratrol anti-fatigue target genes, including Gene ontology (GO) and Kyoto encyclopedia of gene and genome (KEGG) pathway analysis. GO analysis includes analysis of three aspects: cellular component (CC), molecular function (MF), and biological process (BP).

### Molecular docking

PDB database to find and download the PDB file of the core target protein, and use Autodock Tools to dehydrate and hydrogenate it. PubChem to download the mol2 file of resveratrol. Molecular docking of resveratrol to core target proteins using Autodock Vina. And use Pymol software to visualize the docking results.

### Animal experiments

#### Experimental apparatus

SPS202F analytical balance (Zhongzhou Electronic Weighing Apparatus Co., Ltd.), Model 1510 Ultrasonic Cleaner (Wolong Instrument), L-530 Cryogenic Centrifuge (Stevia Technology Development Company), various types of standard glass instruments (Beijing Xinweier Glass Instrument Co., Ltd.), UV-2901 UV-Vis Spectrophotometer (HITACHI Company), BS-220 Mindray Automatic Biochemical Analyzer (Wuhan Shengshida Equipment).

#### Reagents and specifications, origin

**Table:** 

Reagent	Specification	Origin
Resveratrol	AR	Refines biotechnology
Trichloroacetic acid	AR	McLean reagents ltd
Glucose	AR	Aladdin reagents
Sodium carboxymethyl cellulose	AR	McLean reagents ltd
Anthrone	AR	Aladdin reagents
Thiourea	AR	Aladdin reagents
Sodium fluoride	AR	Aladdin reagents
Sodium tungstate	AR	McLean reagents ltd
Trichloroacetic acid	AR	McLean reagents ltd
Copper sulfate	AR	Aladdin reagents
Hydroxybiphenyl	AR	Aladdin reagents
Calcium lactate	AR	Aladdin reagents

#### Experimental method

##### Experiment grouping

Healthy male mice (weight 22 ± 2 g) were provided by the Animal Experiment Center of Ningxia Medical University. The animals were kept in separate cages in a standardized rearing room, maintaining a 12-h day-night cycle, the indoor temperature was controlled at (22 ± 2) °C, and the humidity was controlled at (50 ± 5)%; the animals were free to ingest standard feed and water, and adaptively swim for 20 min per day. After 3 days, they were randomly divided into four groups according to their body weight: A quiet control group (NC), B endurance training control group (EC), C endurance training control + Resveratrol group (EC + RES), D endurance training control group + Glucose group (EC + GLU), 30 mice in each group. Appropriate amounts of resveratrol and glucose were prepared with 0.5% sodium carboxymethyl cellulose (CMC) and stored in a refrigerator at 4 °C (1 mg/ml) for later use. Each group of experimental mice was administered daily at 13:00, (10 mg·kg^−1^, 0.01 ml·g^−1^), and the mice in groups A and B were intragastrically administered with an equal volume of 0.5% CMC for 6 consecutive weeks, and weighed daily. Measure and record body weight. All animal experiments were carried out in strict accordance with the “Regulations of the State Council on the Administration of Laboratory Animals” and the “Administrative Measures for Laboratory Animals of Ningxia Medical University”, and were approved by the Laboratory Animal Ethics Committee of Ningxia Medical University. The mice were anesthetized first and then their necks were severed. The killing method belongs to the euthanasia method of experimental animals, which meets the requirements of ethics.

##### Mice endurance training

The mice in groups B, C, and D were given daily progressive swimming exercises in the experimental animal swimming pool, the water depth was 40–50 cm, and the water temperature was 25 ± 2 °C. Train 5 days a week, once a day in the morning and once in the afternoon. Training time is 9:00 am and 3:00 pm to prepare for exhaustive swimming (ES). The amount of training is shown in Table 1.

**Table 1:** Endurance training schedule (unit: minute).

Time	Monday	Tuesday	Wednesday	Thursday	Friday	Saturday	Sunday
First week	20 × 2	20 × 2	25 × 2	25 × 2	30 × 2		
Second week	30 × 2	35 × 2	35 × 2	40 × 2	40 × 2		
Third week	40 × 2	45 × 2	45 × 2	50 × 2	50 × 2		
Fourth week	50 × 2	55 × 2	55 × 2	60 × 2	60 × 2		
Fifth week	60 × 2	65 × 2	65 × 2	70 × 2	70 × 2		
Sixth week	70 × 2	75 × 2	75 × 2	80 × 2	80 × 2	ES	Sampling

##### ES in mice

Before ES, five mice that were good at swimming and five mice that were not good at swimming were screened out, and there were 20 mice in groups B, C, and D, respectively. On the Saturday of the sixth week of the experiment, ES was performed and recorded.

##### Mice sampling

Mice that performed exhaustive swimming were randomly divided into 2 groups with 10 mice in each group. One group swims for 30 min, 7 mice immediately remove their eyeballs for blood, and 3 mice rest for 100 min and then remove their eyeballs for blood. After blood is drawn from each mouse, 80 μl of whole blood is immediately reserved, and the remaining whole blood is centrifuged at 3000 rpm/minutes at a low temperature (4 °C) for 15 min, and the serum was stored in a − 80 °C refrigerator. The other group did not swim and took blood directly, the method was the same as above. The mice were placed in ice block and sterilized with 75% alcohol, and the liver and gastrocnemius muscles were directly taken out. The tissue was washed with normal saline at 4 °C, dried with filter paper, weighed, and quickly frozen in liquid nitrogen.

##### Detection index and its method

This experiment mainly detects the relevant indicators of anti-exercise fatigue. Mainly include liver glycogen (LG), muscle glycogen (MG), blood lactate (BLA), alanine aminotransferase (ALT), and aspartate aminotransferase (AST), urea nitrogen (BUN), creatinine (CREA), uric acid (UA), blood sugar (GLU) of mice.

*Determination of LG and MG by the anthrone-sulfuric acid method* Extraction of LG (MG): Accurately weigh 100 mg of mouse liver tissue and gastrocnemius muscle tissue for three parallel experiments, add 8 ml of trichloroacetic acid (TCA) respectively, homogenize for 2 min, centrifuge at 2000 rpm for 20 min, and keep the clear liquid. Take 1 ml of the supernatant and put it in a 10 ml centrifuge tube (for three parallel experiments), add 4 ml of 95% ethanol to each tube, mix the two liquids thoroughly, cover, and place in a 37–40 °C water bath for 3 h, 3000 rpm/minutes centrifugation for 15 min. The supernatant was discarded, and the centrifuge tube was placed upside down for 10 min. Slowly add 2 ml of distilled water along the tube wall. Vortex the tube to completely dissolve the glycogen.

At this time, add 10 ml of anthrone reagent to each tube and wait for the tube to reach the temperature of cold water, then immerse it in a boiling water bath and heat for 15 min, then transfer it to a cold water bath, under the UV spectrophotometer, at the wavelength of 620 nm, use a blank reagent tube for calibration and zero adjustments, and measure the absorbance. ( Reagent blank: aspirate 2 ml of distilled water; Standard tube: aspirate 0.5 ml glucose standard solution and 1.5 ml distilled water and mix well)

$${\text{LG}}\,\,\left( {{\text{MG}}} \right){\text{/100g}}\,{ = }\,{\text{DU/DS}} \times 0.5 \times {\text{Extract}}\,\,{\text{volume/Liver}}\,\left( {{\text{muscle}}} \right)\,{\text{tissue}}\,\,{\text{weight}} \times 100 \times 0.9,$$

DU: sample tube absorbance; DS: standard tube absorbance.

*Determination of blood lactic acid in mice by ultraviolet spectrophotometry* Add 1.92 ml of 1% NaF solution to a 5 ml centrifuge tube, and accurately aspirate 80 μl of whole blood into the centrifuge tube. Then add 1.5 ml of protein precipitant, and shake vigorously to mix. (Three groups of parallel experiments were set up for each sample to ensure the accuracy of the experimental results), centrifuge at 3000 rpm for 10 min to obtain the supernatant, and operate according to Table 2.

**Table 2:** Operation Guide (unit: ml).

	Blank tube	Standard tube	Assay tube
Precipitant-NaF mixture	0.5	–	–
Lactic acid standard application solution	–	0.5	–
Supernatant	–	–	0.5
4% CuSO_4_	0.1	0.1	0.1
Concentrated sulfuric acid	3	3	3
Mix well, first heat in a boiling water bath for 5 min, then cool in an ice-water bath for 10 min			
1.5% p-Hydroxybiphenyl	0.1	0.1	0.1

Shake well, heat on a constant temperature water bath at 30 °C for 30 min, then put it in a boiling water bath and heat for 90 s. After cooling to room temperature, calibrate and zero a blank tube with a wavelength of 560 nm under a UV spectrophotometer, and read the absorbance value.

$${\text{BLA}}\,\left( {\text{mg/l}} \right)\,{\text{ = A}}_{{{\text{measuring}}\,\,{\text{tube}}}} {\text{/A}}_{{{\text{standard}}\,\,{\text{tube}}}} \, \times 100 \times 10.$$

*Biochemical index determination* The contents of AST, ALT, BUN, CREA, UA, and GLU were determined by the automatic biochemical analyzer.

##### Statistical method

Statistical method SPSS 21.0 software was used for data statistics. The experimental data are expressed in mean ± standard error (x ± s). First, analysis of variance is used. Paired sample *t* test is used when the variance is homogeneous, and *t*’ test is used when the variance is uneven. When P < 0.05, the difference is considered to be statistically significant.

### Ethics approval

After carefully reading the ARRIVE guidelines, I ensure that all experiments in this study are carried out in accordance with the provisions of the guidelines, follow the recommendations in the arrive guidelines, and meet ethical requirements. This study was performed in line with the principles of the “Regulations of the State Council on the Administration of Laboratory Animals”. This animal experiment plan has also been reviewed by the experimental animal welfare ethics committee of the experimental animal center of Ningxia Medical University, and is in line with the principles of animal protection, animal welfare and ethics, as well as the relevant provisions of the national experimental animal welfare ethics.The mice were anesthetized first and then their necks were severed. The killing method belongs to the euthanasia method of experimental animals, which meets the requirements of ethics.

## Results

### Acquisition of anti-fatigue targets of resveratrol

**Figure 1:**
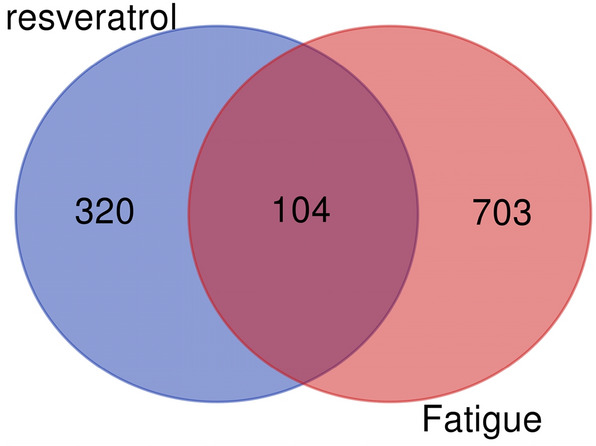
Venn map.

A total of 320 targets for resveratrol and 703 targets for fatigue were obtained. The intersection of the two is the anti-fatigue target of resveratrol, with a total of 104 targets, as shown in Fig. 1.

### PPI network and screening of core target proteins

**Figure 2:**
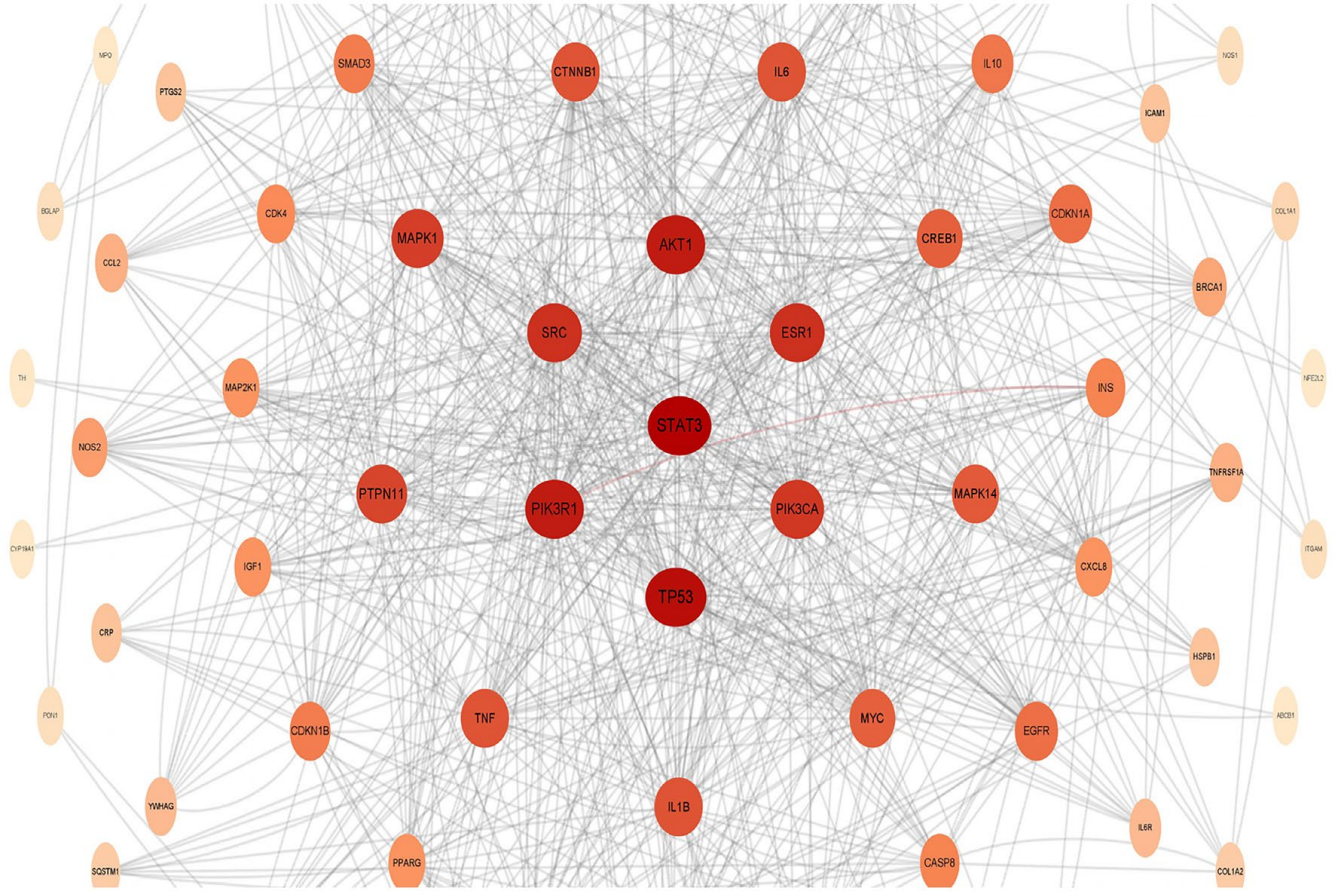
PPI network map.

The PPI network diagram of resveratrol anti-fatigue targets is shown in Fig. 2. A total of 104 nodes and 804 edges are included. The core target proteins were screened according to the topological parameters moderate centrality, compact centrality, and mediation centrality. The proteins with larger values were STAT3, TP53, PIK3R1, AKT1, SRC, ESR1, PIK3CA, MAPK1, PTPN11, and CTNNB1. The topological parameters of resveratrol anti-fatigue core target genes are shown in Table 3.

**Table 3:** Topological parameters of resveratrol anti-fatigue core target genes.

Target	Moderate centrality	Compact centrality	Mediation centrality	Clustering coefficient
STAT3	0.1016	0.5116	0.1016	0.2710
TP53	0.1108	0.5000	0.1108	0.2389
PIK3R1	0.07581	0.5176	0.1164	0.2792
AKT1	0.1164	0.5207	0.0758	0.2906
SRC	0.0359	0.4835	0.0666	0.3297
ESR1	0.0666	0.4862	0.0359	0.3659
PIK3CA	0.04163	0.5087	0.04163	0.3636
MAPK1	0.0479	0.4862	0.0479	0.2814
PTPN11	0.0362	0.4862	0.03617	0.3952
CTNNB1	0.0176	0.4706	0.0625	0.2865

### GO enrichment analysis

**Figure 3:**
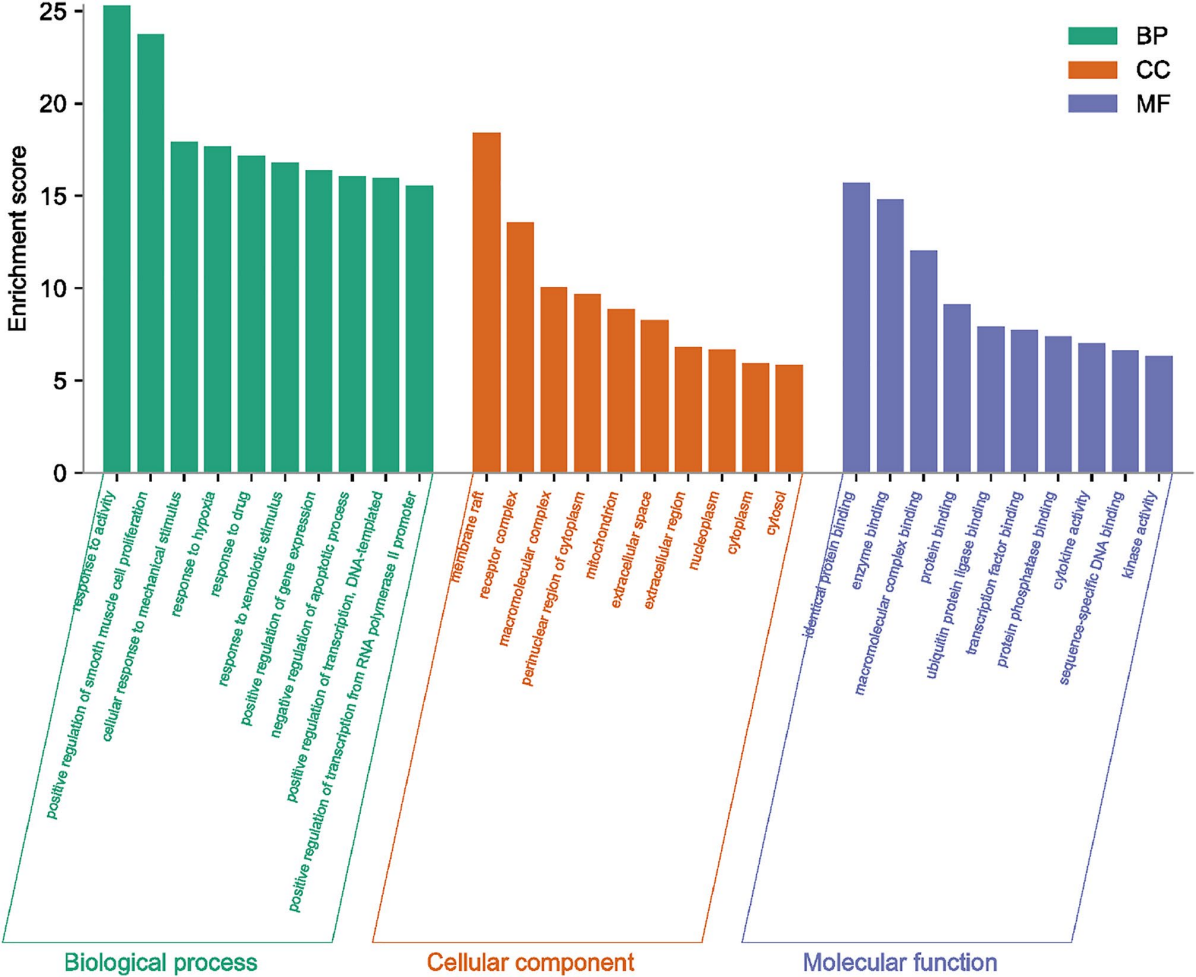
GO enrichment analysis.

The GO enrichment analysis results of resveratrol anti-fatigue targets are shown in Fig. 3. The GO analysis includes three aspects: BP, CC, and MF. BP mainly involves response to activity, positive regulation of smooth muscle cell proliferation, cellular response to mechanical stimulus, response to hypoxia, etc. CC mainly focuses on membrane raft, receptor complex, macromolecular complex, perinuclear region of cytoplasm, etc. MF mainly involves the identical protein binding, enzyme binding, macromolecular complex binding, protein binding, etc.

### KEGG enrichment analysis

**Figure 4:**
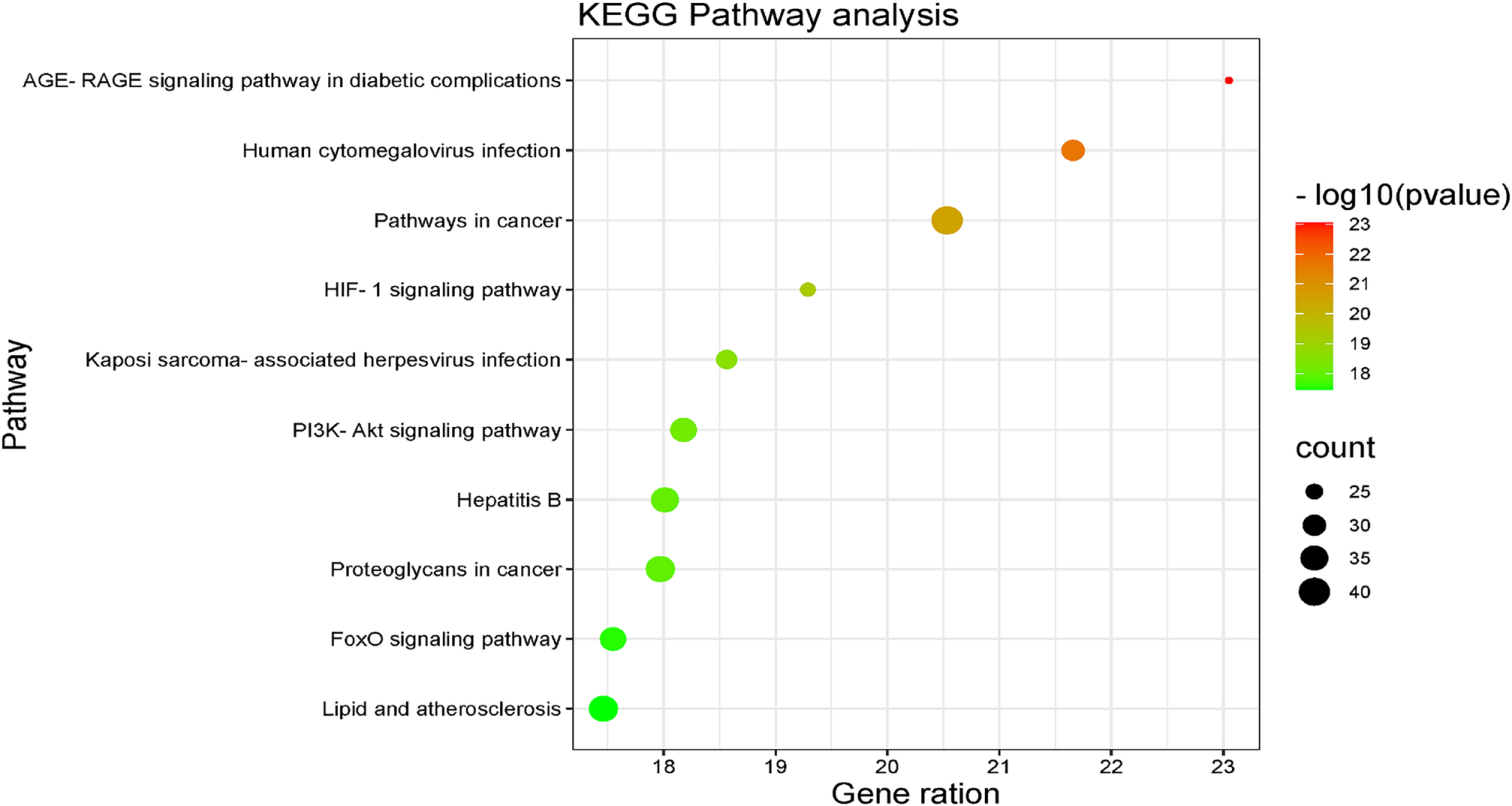
KEGG enrichment analysis.

The KEGG enrichment analysis results of resveratrol anti-fatigue targets are shown in Fig. 4. Pathways with higher enrichment significance mainly include the AGE-RAGE signaling pathway in diabetic complications (AGE-RAGE), Human cytomegalovirus infection (HCI), Pathways in cancer (PIC), and so on.

**Figure 5:**
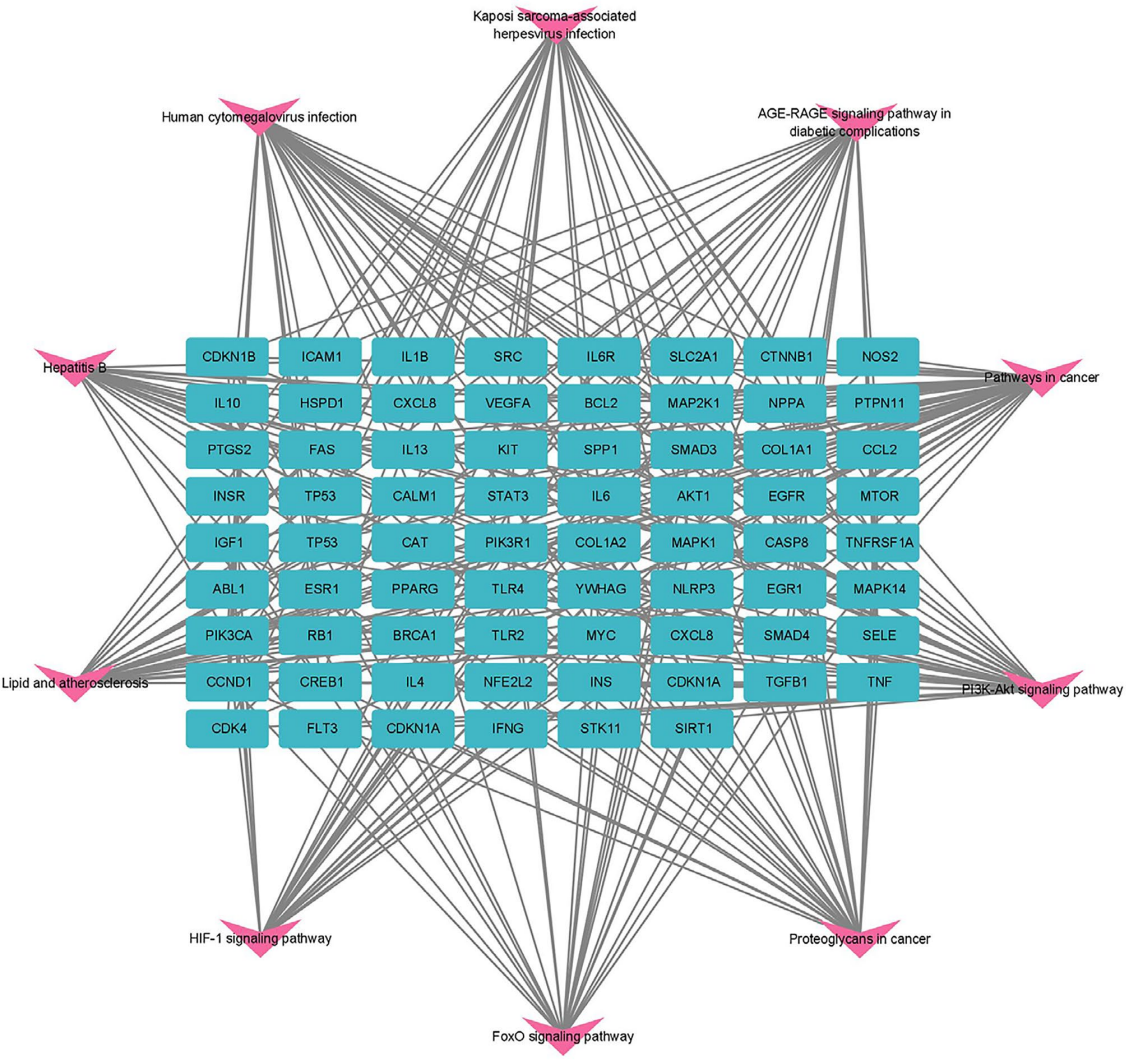
Network diagram of the relationship between target genes and the KEGG pathway.

The network diagram of the relationship between target genes and the KEGG pathway is shown in Fig. 5. It can be seen from the figure that the target genes enriched together between the core pathway and other pathways.

**Figure 6:**
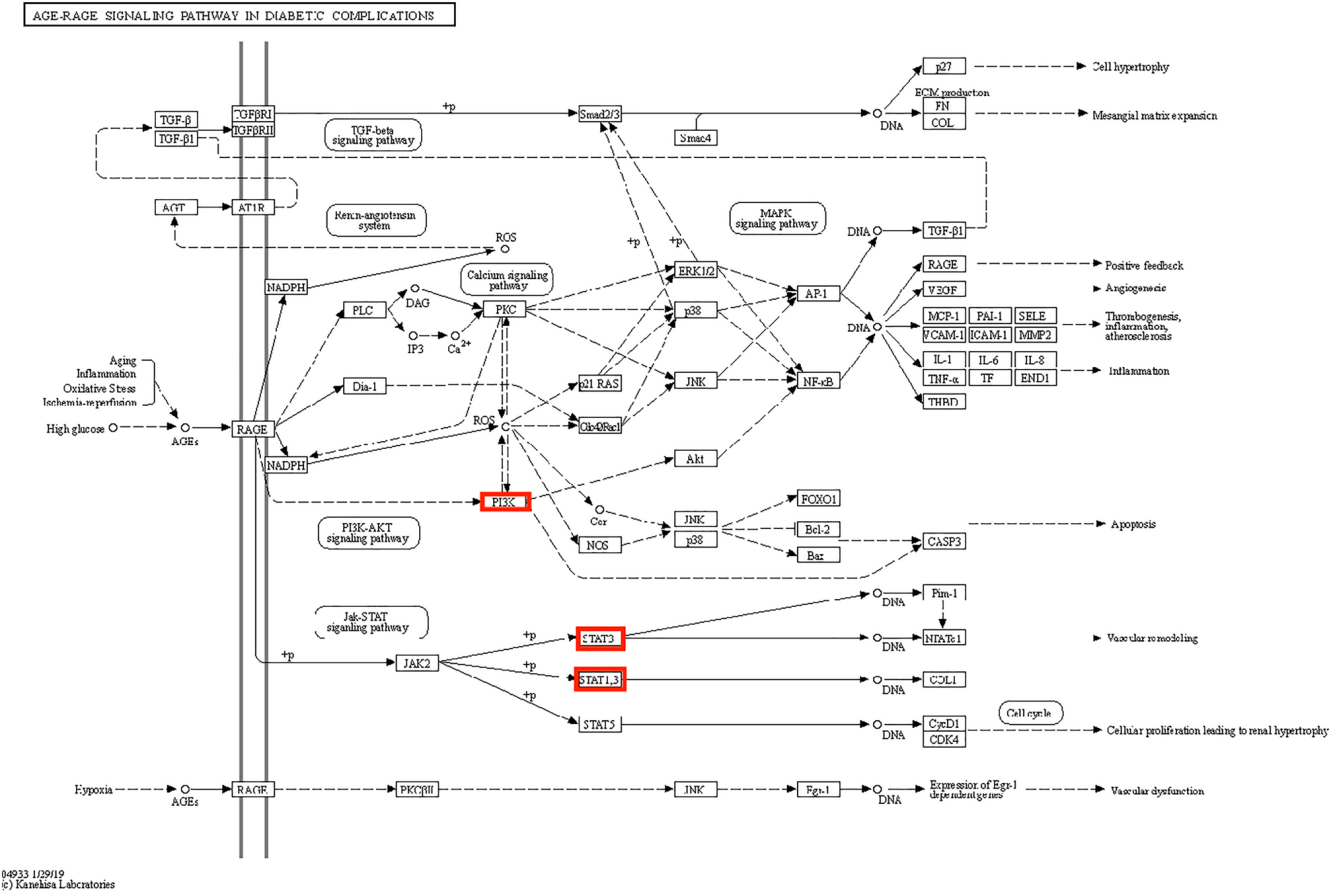
The location of core target genes in AGE-RAGE. Figure 6 is available on the website at https://www.kegg.jp/pathway/map04933.

**Figure 7:**
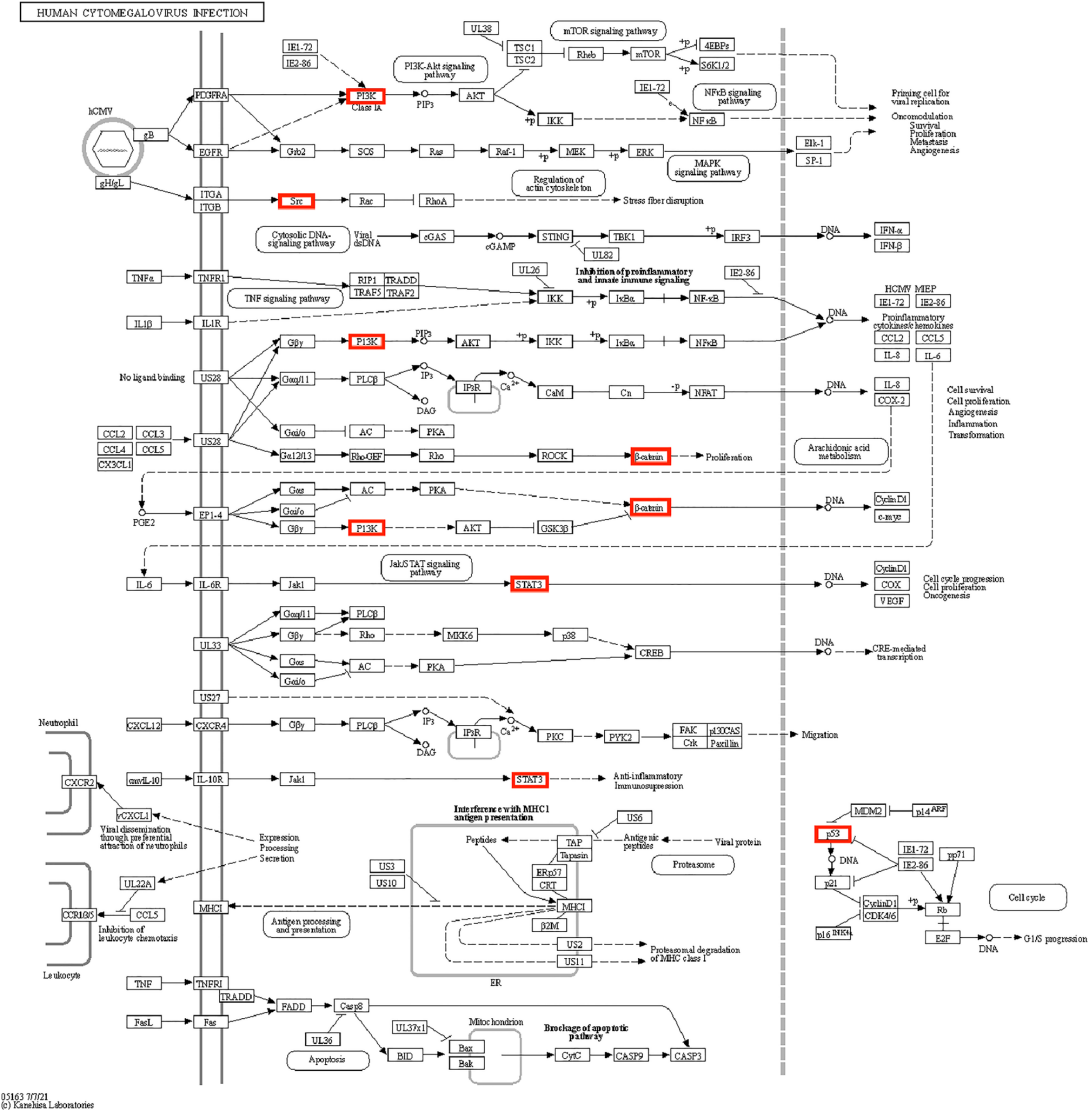
The location of core target genes in HCI. Figure 7 is available on the website at https://www.kegg.jp/pathway/map05163.

The positions of core target genes in AGE-RAGE and HCI are shown in Figs. 6 and 7.

### Molecular docking results between resveratrol and the protein encoded by the core target gene

Resveratrol was molecularly docked with the core target protein, and the binding energy data results are shown in Table 4.

**Table 4:** Molecular docking results of resveratrol and core target protein.

Core target protein	Binding energy/(kJ∙mol^−1^)
STAT3	− 4.58
TP53	− 5.14
PIK3R1	− 5.14
AKT1	− 5.97
SRC	− 4.69
ESR1	− 4.49
PIK3CA	− 5.11
MAPK1	− 5.03
PTPN11	− 3.62
CTNNB1	− 4.14

**Figure 8:**
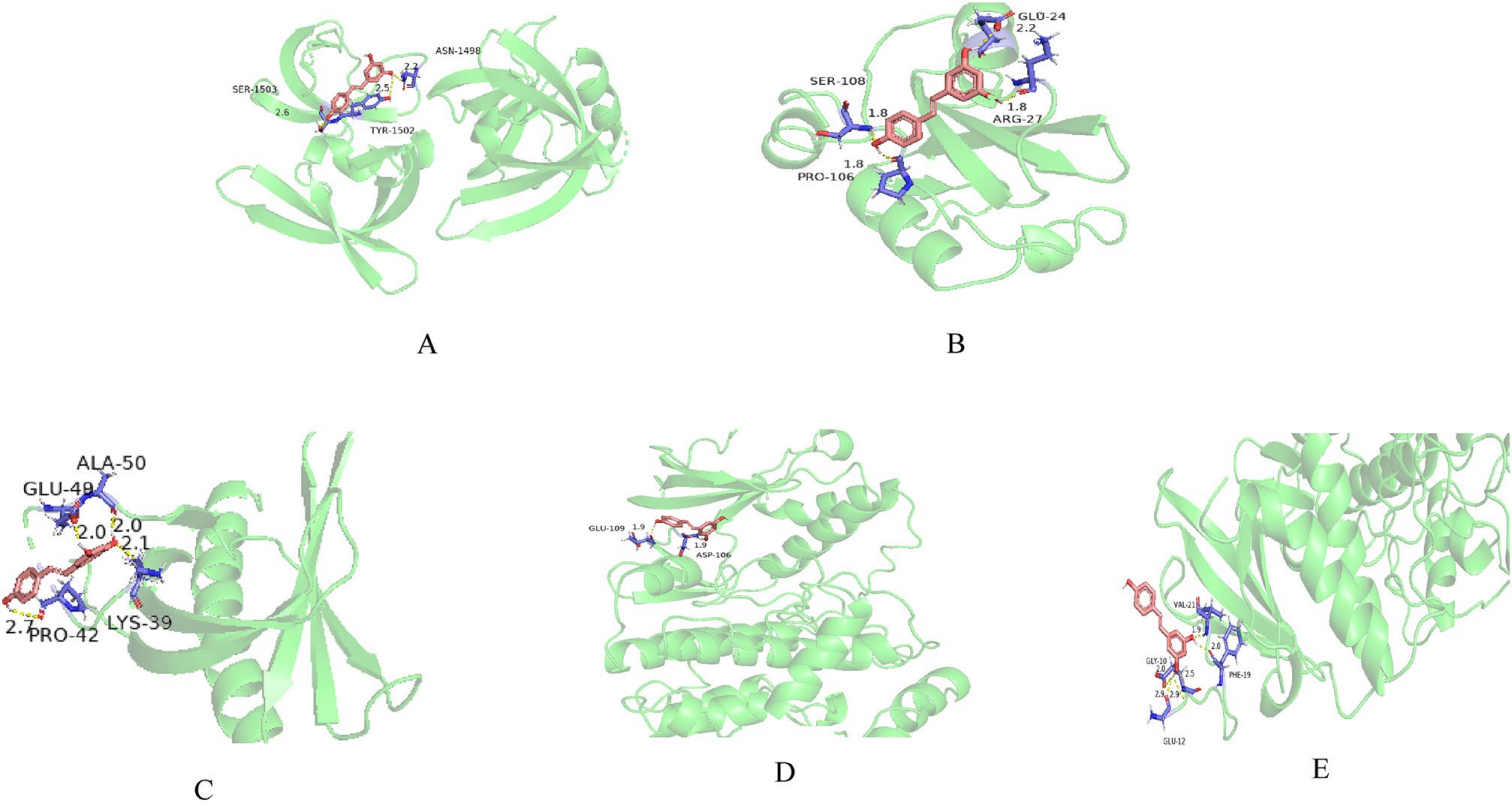
Schematic diagram of molecular docking between resveratrol and core target protein, (**A**) TP53-Resveratrol, (**B**) PIK3R1-Resveratrol, (**C**) AKT1-Resveratrol, (**D**) PIK3CA- Resveratrol, (**E**) MAPK1-Resveratrol.

It is generally considered that if the binding energy < − 5.0 kJ/mol, it indicates that this substance has a good binding ability to the docking target protein. In this study, 5 of the 10 core target proteins have binding energies to resveratrol < − 5.0 kJ/mol, indicating that these 5 core proteins bind well to resveratrol. The schematic diagram of the molecular docking between core protein < − 5.0 kJ/mol and resveratrol is shown in Fig. 8.From the figure, we can see the specific docking position and bond length between resveratrol and core protein.

### Animal experiments

#### Results of exhaustive swimming in experimental mice

A quiet control group (NC), B endurance training control group (EC), C endurance training control + Resveratrol group (EC + RES), D endurance training control group + Glucose group (EC + GLU).

Table 5, resveratrol intervention can prolong the exhaustive swimming time of mice (P < 0.01). The length of swimming time can directly reflect the fatigue degree of mice Therefore, resveratrol can improve fatigue symptoms of endurance training mice, enhance exercise ability of mice and delay the occurrence of exercise fatigue.

**Table 5:** Exhaustive swimming time (unit: minute).

Group	ES
NC	189 ± 37
EC	237 ± 88*
EC + RES	284 ± 70*
EC + GLU	254 ± 96

Compared with NC group, *means P < 0.01.

#### Indicator test results

Table 6, resveratrol intervention can increase LG and MG (P < 0.01),and decrease BLA (P < 0.01) in mice. Explain that resveratrol can delay fatigue and improve exercise ability of endurance training mice.

**Table 6:** Biochemical parameters.

Group	LG (mg/g)	MG (mg/g)	BLA (mmol/l)
NC	2.4 ± 1.5	1.6 ± 1.4	3.1 ± 1.2
EC	2.1 ± 1.4	1.4 ± 1.3	7.9 ± 1.5
EC + RES	4.9 ± 1.3^▪^	2.1 ± 1.4^▪^	5.3 ± 1.4^▪^
EC + GLU	2.8 ± 1.5	1.8 ± 1.4	7.4 ± 1.3

Compared with NC group, ▪means P < 0.01.

Table 7, resveratrol could decrease AST, ALT, UA (P < 0.01) and BUN (P < 0.05) in mice. Although it can reduce CREA of mice (P > 0.05), the effect is not obvious. Resveratrol could also reduce Glu depletion in mice (P > 0.05), but the effect was not obvious. Explain that resveratrol has antifatigue effect.

**Table 7:** Blood biochemical parameters (unit: mmol/l).

Group	AST	ALT	BUN	CREA	UA	GLU
NC	111.1 ± 1.3	45.2 ± 1.3	6.0 ± 1.5	28.1 ± 1.5	104.1 ± 1.3	8.1 ± 1.3
EC	119.2 ± 1.2	50.1 ± 1.3	7.4 ± 1.5	27.2 ± 1.3	138.1 ± 1.3	6.1 ± 1.2
EC+RES	107.1 ± 1.4^**^	48.1 ± 1.2^**^	6.9 ± 1.4^*^	26.2 ± 1.4	123.2 ± 1.2^**^	7.2 ± 1.4
EC+GLU	127.2 ± 1.4	49.2 ± 1.1	7.0 ± 1.5	28.3 ± 1.2	137.2 ± 1.4	7.2 ± 1.2

Compared with NC group, ^*^means P < 0.05, ^**^means P < 0.01.

## Discussion

Under today’s fast-paced living conditions, unreasonable diet, lack of exercise, irregular work and rest, mental stress, high psychological pressure, and long-term negative emotions are prevalent^13,14^. Therefore, more and more people are in a sub-health state and face “unexplained fatigue”. If the treatment is inappropriate, recurrent fatigue may transfer into chronic fatigue^15^, which can bring a lot of troubles to people’s physical and psychological health. As many as one-third of adults have been reported to experience chronic fatigue for 6 months or more. Fatigue is a complex and comprehensive physiological phenomenon with no clear etiology and may require long-term medical treatment. Experts and scholars have been devoted to the research on the effective prevention and treatment of fatigue, but there are still many problems that remain unclear. Resveratrol has a variety of biological functions and medicinal values^16^. Both in vivo and in vitro studies have confirmed that resveratrol is a strong oxidant with a high scavenging ability to various free radicals and can effectively inhibit lipid peroxidation^17^. During high-intensity exercise, the body’s metabolism is enhanced and oxygen consumption increases, increasing the level of free radicals, and free radicals can damage the mitochondrial respiratory chain to produce adenosine triphosphate (ATP), resulting in cellular energy synthesis and muscle fiber contraction dysfunction^18^. Given the rich biological effects of resveratrol, this study speculates that resveratrol can improve the body’s exercise capacity and delay fatigue. Therefore, it is necessary to use network pharmacology to predict targets and analyze drug pathways to explore the mechanism of resveratrol in anti-fatigue. And in vitro animal experiments were used to further verify the anti-fatigue effect of resveratrol. In this study, 104 common targets between resveratrol and fatigue were predicted, the PPI network map of resveratrol’s anti-fatigue targets was constructed, and the core target proteins were screened, namely STAT3, TP53, PIK3R1, AKT1, SRC, ESR1, PIK3CA, MAPK1, PTPN11, and CTNNB1. These targets are mainly concentrated in cell proliferation, apoptosis, inflammation, and so on. These core targets play an important role in resveratrol’s anti-fatigue process. Subsequently, in this study, GO enrichment analysis was performed on the anti-fatigue targets of resveratrol, and the types mainly involved in MF, the areas where CC was mainly concentrated, and the reactions mainly involved in BP were obtained. KEGG enrichment analysis obtained pathways with higher enrichment significance. The affinity between resveratrol and core target protein was verified by molecular docking, and the results showed that resveratrol had a good binding effect with TP53, PIK3R1, AKT1, and PIK3CA, and MAPK1. In addition, this study confirmed through animal experiments that resveratrol could significantly prolong the exhaustive swimming time of endurance-trained mice (P < 0.01). At the same time, in the detection of biochemical indicators, it was found that resveratrol could reduce AST (P < 0.01), ALT (P < 0.01), and UA (P < 0.01) in endurance training mice, and at the same time could increase LG and MG (P < 0.01), decreased BUN (P < 0.05), and decreased BLA (P < 0.01). Based on the above studies, resveratrol can improve the exercise ability of endurance training mice and has an anti-fatigue effect.

## Conclusion

This study used network pharmacology to analyze the anti-fatigue mechanism of resveratrol from the two aspects of action target and action pathway. Resveratrol may exert its anti-fatigue effect by acting on core target genes such as TP53, PIK3R1, AKT1, PIK3CA and MAPK1, thereby affecting the AGE-RAGE, HCI and PIC pathways. Resveratrol has multiple targets and multiple pathways. The anti-fatigue characteristics of resveratrol provide a theoretical basis for the application of resveratrol in anti-fatigue. The anti-fatigue efficacy of resveratrol was further verified through animal experiments. The results showed that resveratrol could improve the exercise capacity of endurance training mice and had the effect of delaying fatigue, which provided a new direction for the development of anti-fatigue preparations or health products.

## Data Availability

All data generated or analysed during this study are included in this published article.
